# Effects of feeding a *Saccharomyces cerevisiae* fermentation product compared to a direct-fed microbial in finishing diets of beef × dairy crossbred steers fed in the Pacific Northwest

**DOI:** 10.1093/tas/txaf098

**Published:** 2025-07-22

**Authors:** Sydney M Bowman-Schnug, Bradley J Johnson, O Abe Turgeon, Joaquin Figueroa, Craig R Belknap, Zebadiah T L Gray, Thomas S Edrington

**Affiliations:** Department of Animal and Food Sciences, Texas Tech University, Lubbock, TX 79409, USA; Department of Animal and Food Sciences, Texas Tech University, Lubbock, TX 79409, USA; Turgeon Consulting Service, LLC, Amarillo, TX 79118, USA; Beef Northwest Research, Pendleton, OR 97801, USA; Diamond V, Cedar Rapids, IA 52404, USA; Diamond V, Cedar Rapids, IA 52404, USA; Diamond V, Cedar Rapids, IA 52404, USA

**Keywords:** beef × dairy, feedlot cattle, fermentation product, finishing, health, *Saccharomyces cerevisiae*

## Abstract

The objective of this study was to evaluate the effects of a *Saccharomyces cerevisiae* fermentation product (SCFP) compared to a direct-fed microbial (DFM) on growth performance, health, carcass characteristics, and liver abscess prevalence in beef × dairy crossbred steers. Two thousand steers [50% beef, 25% Holstein, 25% Jersey genetics; initial shrunk body weight (SBW) = 288.2 ± 8.0 kg] were blocked by arrival date and randomly assigned to receive 1 of 2 treatments: 1) SCFP supplied in the starter diet at 12 g per steer daily and then 9 g per steer daily in the finishing diet (NS; NaturSafe™, Diamond V, Cedar Rapids, IA) or 2) DFM fed at 50 mg per steer daily throughout the feeding period (BD; Bovamine Defend, Chr. Hansen, Milwaukee, WI). Pen served as the experimental unit (200 steers/pen), with 5 pens per treatment. Data were analyzed as a randomized complete block design in R 4.2.2. with the main effect of treatment and random effect of block included in the model. Results were reported on a deads-in basis unless otherwise stated. Cattle were fed for a total of 275 ± 6.2 d. Initial and final SBW did not differ (*P* ≥ 0.84) by treatment. Initial treatment pulls were observed more frequently for NS compared to BD cattle (29.43% vs. 21.67%; *P* < 0.01). However, NS cattle had a lesser rate of repulls as a proportion of initial pulls (10.08% vs. 16.61%; *P* = 0.03). Fewer (*P* < 0.01) bullers were reported amongst NS cattle. Cattle supplemented with NS had a lower case fatality rate (6.08% vs. 11.96%; *P* < 0.01) and tended to have a lower total mortality rate (1.60% vs. 2.70%; *P* = 0.09) than BD. With deads included, average daily gain (ADG) tended (*P* = 0.06) to be greater for NS cattle. Dry matter intake did not differ (*P* = 0.99) by treatment; however, NS cattle had a numeric advantage in feed efficiency (G:F) nearing a tendency (0.132 vs. 0.130; *P* = 0.11). On a deads-out basis, ADG and G:F were similar (*P *≥ 0.85). Dressing percentage tended (*P* ≤ 0.10) to be greater for NS carcasses. Cattle fed BD had a greater (*P* = 0.03) proportion of USDA Prime carcasses. While treatment had no impact on liver abscess severity or total abscess occurrence, NS cattle tended to have less A- abscesses (1.72% vs. 3.87%; *P* = 0.10). In this large-pen comparison, SCFP supplementation improved feedlot cattle health and positively influenced performance compared to a DFM.

## Teaser

There is potential the *Saccharomyces cerevisiae* fermentation product (SCFP) provided immune support for morbid cattle, allowing them to recover more rapidly and with less outside intervention. Additionally, a lower case fatality rate suggests fewer morbid cattle supplemented with NaturSafe (NS) succumbed to their ailment, providing further evidence supporting SCFP-modulated improvements in cattle health and immunity.

## INTRODUCTION

Alternative feed additives, such as direct-fed microbials (DFMs) and probiotics, have gained increasing attention over the last several decades in the cattle feeding industry. Dietary inclusion is most often associated with antimicrobial replacement in natural feeding programs. However, conventional systems may also benefit from potential improvements in health and performance. *Saccharomyces cerevisiae* fermentation products (SCFP) contain bioactive compounds capable of supporting such results. Immunomodulatory properties of SCFP have been shown to support health improvements in feedlot cattle (Zinn et al., 1999; Jensen et al., 2008; Rients et al., 2023). Additionally, SCFP have been reported to mitigate digestive disturbances, which are associated with the ability of the yeast culture to stabilize the rumen environment (Callaway and Martin, 1997; Moya et al., 2009). This benefit is especially important when feeding dairy-derived cattle, as extended days on feed and greater dry matter intake (DMI) put them at increased risk of metabolic disorders (Duff and McMurphy, 2007). Health challenges of any kind are detrimental to cattle performance (Anderson and Gleghorn, 2007), and provision of SCFP has been linked to improved cattle gain and efficiency (Hinman et al., 1998; Wagner et al., 2016).

With such critical impacts on growth performance, SCFP are of particular interest for dietary inclusion in feedlot diets. However, there are numerous alternative feed additives marketed for similar benefits in the cattle feeding industry. One such product category with extensive tenure is DFM. Direct-fed microbials contain live or naturally occurring organisms that are believed to favorably alter ruminal environments, and many of these products have also reported positive effects on animal performance, health, and pathogen exclusion (McAllister et al., 2011; Thompson et al., 2020; Guimares et al., 2024). With similar reported traits, comparison of product efficacy is of particular interest in evaluating production use. Therefore, the objective of this study was to evaluate the effects of a SCFP compared to a DFM on growth performance, health, carcass characteristics, and liver abscess prevalence in finishing diets of beef × dairy crossbred steers.

## MATERIALS AND METHODS

### Animal Care and Use

The following was a collaborative experiment conducted at the Beef Northwest Stage Gulch Research Facility in Pendleton, OR. All research followed the guidelines stated in the Guide for the Care and Use of Agricultural Animals in Agricultural Research and Teaching (FASS, 2020)  .

### Animals, Diet, and Experimental Design

A total of 2000 beef × dairy crossbred steers (50% beef, 25% Holstein, 25% Jersey genetics) with an initial shrunk body weight (SBW) of 288.2 ± 8.0 kg were obtained from 2 origins. Cattle were blocked by arrival time (5 blocks; 400 steers per block; 2 pens per block) and assigned to pens on an every-other animal basis prior to initial processing. Arrival pen weights were recorded using a mobile scale (Rice Lake Livestock Scales; Rice Lake, WI) and shrunk by 4% to determine initial SBW. At processing, steers were also individually weighed (Silencer Hydraulic Squeeze Chute; Moly MFG Inc., Lorraine, KS), received a pour-on for internal and external parasite control (Bimectin Pour-On; Bimeda, Oakbrook Terrace, IL), vaccinated (Pyramid 10, Boehringer Ingelheim Animal Health, Duluth, GA), and administered an ear implant (Synovex One Feedlot; Zoetis Inc., Kalamazoo, MI). Additionally, cattle received a unique electronic identification tag (Merck Animal Health, Rathway, NJ) and a drop down ear tag to identify origin and lot (Temple Tags; Temple, TX).

Pens were randomly assigned to 1 of 2 treatments (*n *= 5 pens per treatment; 200 steers per pen) in a randomized complete block design. Treatments started at arrival and pens of cattle were assigned to receive either: 1) SCFP (NaturSafe, Diamond V Mills, Inc., Cedar Rapids, IA) supplied in the starter and transition diets at 12 g per steer daily and then at 9 g per steer daily in the finishing diet (NS) or 2) DFM formulated to provide a minimum of 2 × 10^13^ CFU of *Lactobacillus animalis* and *Propionibacterium freudenreichii* (Bovamine Defend, Chr. Hansen, Milwaukee, WI) supplied at 50 mg per steer daily throughout the entire feeding period (BD). For application of the treatments to the first 2 blocks (4 pens total), the necessary inclusion of Bovamine Defend and NaturSafe were mixed thoroughly with cull French fries in 5-gallon buckets and top-dressed on the first feeding. This process was repeated daily until enough cattle arrived to ensure adequate load size, at which point treatments were able to be delivered using the Micro-Weigh System (Micro Technologies, Amarillo, TX).

Diet compositions and formulated analyses for the receiving diet are shown in Table 1. Micro ingredients supplied in the receiving diet included Rumensin (27.6 mg/kg; Elanco, Greenfield, IN), Tylan (75 mg/hd/d; Elanco, Greenfield, IN), Availa 4 (7 g/hd/d; ZinPro, Eden Prairie, MN), and vitamins A (100,000 IU/hd/d), D (10,000 IU/hd/d), and E (500 IU/hd/d). The receiving diet was fed for 5 to 7 d before initiation of the two-ration blend program used during the transition period. Four steps, between 3 to 5 d in length each, were utilized in which a decreasing proportion of the receiving diet and an increasing proportion of the finishing diet were supplied to steers until they were solely consuming the finisher. Diet compositions and formulated analyses in the finishing diet are shown in Table 2. Micro ingredients supplied in the finishing diet included Rumensin (48.9 mg/kg; Elanco, Greenfield, IN), Tylan (80 to 90 mg/hd/d; Elanco, Greenfield, IN), Availa Zn (5 g/hd/d; ZinPro, Eden Prairie, MN), and vitamins A (50,000 IU/hd/d), D (5,000 IU/hd/d), and E (50 IU/hd/d). Diets were reformulated periodically throughout the feeding period based on feedstuff availability, diet cost, and fluctuating nutrient concentrations. Dietary energy density was kept consistent between each diet formulation. The amount of time that cattle received each formulation was kept consistent within block.

**Table 1.** Formulated dry matter ingredient inclusion in the basal receiving diet, which was supplied to cattle of both treatments

	Formulation Sequence^1^	
	1^*^	2
**Date of Formulation**	07/12/2022	07/18/2022
**Formulated DM inclusion** ^2^ **, %**		
Steam-flaked corn	36.70	33.90
Alfalfa hay	35.80	35.90
Hopper^3^	11.80	12.80
Cull French fries	9.00	10.80
Wet distillers grain	4.20	4.20
Liquid finisher supplement	2.50	2.40
**Chemical Analysis**		
Dry matter, %	55.30	58.50
Crude protein, %	14.60	14.50
Non-protein nitrogen, %	1.90	1.60
Crude fiber, %	12.80	12.80
Crude fat, %	4.70	5.00
Ca, %	0.90	0.90
P, %	0.30	0.30
K, %	1.40	1.40
S, %	0.20	0.20
Na, %	0.08	0.08
Cl, %	0.12	0.12
NE_M_, Mcal/kg	1.81	1.81
NE_G_, Mcal/kg	1.10	1.10

^*^For the first 2 blocks of cattle only, Bovamine Defend (50mg/hd/d) and NaturSafe (12 g/hd/d) were mixed thoroughly with processed potato waste in a 5-gal bucket and top dressed on the first feeding until enough cattle arrived to ensure adequate load size so that treatments were able to be delivered using the Micro-Weigh System (Micro Technologies; Amarillo, TX).

^1^Formulation sequence references only the order in which each subsequent diet was formulated. Diets were reformulated periodically to align with commodity availability and to keep dietary energy density consistent.

^2^Receiving diets were additionally supplemented with 27.6 mg/kg DMB Rumensin (Elanco, Greenfield, IN), 75 mg/hd/d Tylan (Elanco, Greenfield, IN), 7 g/hd/d Availa 4 (ZinPro, Eden Prairie, MN), 100,000 IU/hd/d vitamin A, 10,000 IU/hd/d vitamin D, and 500 IU/hd/d vitamin E.

^3^Hopper is slices, pieces, and chunks of unprocessed potatoes.

**Table 2.** Formulated dry matter ingredient inclusion in the basal finishing diet, which was supplied to cattle of both treatments

	Formulation Sequence^1^				
	1	2	3	4	5
Date of Formulation	07/18/2022	11/03/2022	12/20/2022	02/20/2023	06/03/2023
**Formulated DM inclusion** ^2^ **, %**					
Steam-flaked corn	45.10	36.00	36.90	35.40	39.70
Alfalfa hay	8.00	10.00	10.00	10.00	8.00
Hopper^3^	17.30	17.90	16.50	17.60	20.60
Cull French fries	20.70	24.40	24.30	25.80	20.80
Wet distillers grain	4.90	7.70	7.90	6.70	6.40
Liquid finisher supplement	4.00	4.00	4.40	4.50	4.50
**Chemical Analysis**					
Dry matter, %	51.90	43.60	40.10	42.60	44.30
Crude protein, %	13.00	13.00	13.00	13.00	13.00
Non-protein nitrogen, %	2.70	2.70	2.90	3.00	3.00
Crude fiber, %	5.30	6.00	5.90	5.60	4.70
Crude fat, %	7.50	7.50	7.50	7.50	7.50
Ca, %	0.70	0.80	0.80	0.80	0.80
P, %	0.30	0.30	0.30	0.30	0.30
K, %	0.90	1.10	1.00	1.10	1.00
S, %	0.20	0.20	0.20	0.20	0.20
Na, %	0.12	0.12	0.12	0.12	0.12
Cl, %	0.18	0.18	0.18	0.18	0.18
NE_M_, Mcal/kg	2.12	2.10	2.10	2.11	2.09
NE_G_, Mcal/kg	1.41	1.40	1.39	1.40	1.39

^1^Formulation sequence references only the order in which each subsequent diet was formulated. Diets were reformulated periodically to align with commodity availability and to keep dietary energy density consistent.

^2^Finishing diets were additionally supplemented with 48.9 mg/kg DMB Rumensin (Elanco, Greenfield, IN), 80 to 90 mg/hd/d Tylan (Elanco, Greenfield, IN), 5 g/hd/d Availa Zn (ZinPro, Eden Prairie, MN), 50,000 IU/hd/d vitamin A, 5,000 IU/hd/d vitamin D, and 50 IU/hd/d vitamin E.

^3^Hopper is slices, pieces, and chunks of unprocessed potatoes.

Cattle were stocked at approximately 14.9 square meters of pen space per animal. Each pen also included 24.4 cm of concrete bunk space per animal and a water trough. Steers were fed three times per day. Feed bunks were managed to a slick/clean bunk management system with content cattle that were not aggressive at first feeding. Thirty minutes prior to first feeding, bunk condition and animal behavior were evaluated and feed calls were completed by trained personnel. Feed bunks and cattle were monitored for re-feeds daily. Samples of each diet and all feed ingredients were taken daily and dried in a forced-air oven at 105 °C for 24 hours to determine dry matter percentages. These percentages were updated daily in the bunk reader system to ensure the accuracy and quality of feed calls. Additionally, feed samples were sent twice monthly to a commercial laboratory (SDK Laboratories, Hutchinson, KS) for chemical analysis.

Cattle health and general well-being was evaluated daily by trained pen riders, with pulls being made for symptoms including dull eyes, droopy ears, anorexia, nasal discharge, labored breathing, lameness, and diarrhea. The pull, treat, return (PTR) system was utilized for steers identified as sick or injured. Steers were moved to the nearest treatment facility, where body weight was recorded and rectal temperature was taken using a digital thermometer. All diagnoses were made by trained personnel and treatment plans were executed according to feedlot protocol prescribed by the consulting veterinarian. Respiratory ailments were treated initially with tulathromycin and ketoprofen (Draxxin KP, Zoetis Inc., Kalamazoo, MI). Cattle requiring a secondary treatment received florfenicol (Nuflor, Merck Animal Health, Rahway, NJ), and animals requiring a third treatment were administered tildipirosin (Zuprevo, Merck Animal Health, Rahway, NJ). All animals diagnosed with lameness were treated with ceftiofur crystalline free acid (Excede, Zoetis Inc., Kalamazoo, MI). Gaseous bloat was treated using poloxalene (Therabloat, Zoetis Inc., Kalamazoo, MI) and a laxative (MagLax, MWI Animal Health, Boise, ID). Once evaluated and treated accordingly, steers were returned to their home pen the same day. Animals requiring further observation remained in 1 of 4 hospital pens (2 pens per treatment), where they received their appropriate treatment diet. Chronically ill or unthrifty cattle were marketed as railers and were included in the database.

Approximately 60 d prior to their anticipated ship date, a terminal implant and terminal sort was performed. Steers were reimplanted (Synovex Choice, Zoetis, Kalamazoo, MI), vaccinated for respiratory (Pyramid 3 + Presponse, Boehringer Ingelheim Animal Health, Duluth, GA) and clostridial diseases (Ultrachoice 7, Zoetis Inc., Kalamazoo, MI), received a pour-on for parasite control (Permectrin CDS Pour-ON, Elanco, Greenfield, IN) and a probiotic drench (Lactipro NXT, Axiota, Fort Collins, CO). At the same time, the PenPoint Sort System (Elanco, Greenfield, IN) was utilized to sort cattle into three outcome market groups. Sort groups and individual animals were identified with a notch on their drop-down ear tag. All cattle were then returned to their original home pen. Pen riders sorted outcome groups directly out of their home pen on shipment days based on their notched ear tag.

On their predetermined ship date, cattle were transported approximately 72.4 km for harvest at Tyson (Pasco, WA). Cattle were fed normally the day before shipment. Those that were shipped before 12:00 p.m. were not fed the morning of harvest. All cattle that shipped after 12:00 p.m. were fed during the first round of morning feeding, receiving a third of their previous day’s feed call. Time of shipment was kept consistent between treatments by block.

### Measures of Health Response

Health data were collected throughout the feeding period. Costs associated with treatment, including medicine costs, were recorded and analyzed. Initial pulls were defined as all cattle removed from their home pen and transported to a hospital because of suspected disease or defect. Repulls were those cattle pulled again for further treatment. Neither initial pulls nor repulls included bullers. Bullers were animals expressing excessive riding or imitating bull-like behavior. Railers were identified as steers that appeared unthrifty or chronically ill and were deemed unfit to market with their pen mates. This included cattle that were unresponsive to therapy after three respiratory treatments, animals that were lame, crippled, or injured, and individuals that were clearly behind the average pen weight and likewise unmerchantable within that pen. All mortalities were recorded, and necropsies were performed by trained personnel via the consulting veterinarian to determine cause of death. Day on feed at the time of mortality was additionally recorded and each mortality was categorized chronologically as early in the feeding period, occurring within the first 45 d on feed; mid-feeding period; or late in the feeding period, occurring in the last 90 d on feed.

### Feedlot Performance Measures

Steers were weighed upon arrival to determine their initial SBW. Daily feed deliveries were recorded and cross-analyzed with steer inventories to determine daily dry matter intake (DMI). Railers remained in their home pens on their assigned treatments until being shipped to ensure accurate accounting of all DMI. Steers were weighed prior to shipping for harvest and a predetermined shrink was applied to calculate final shrunk BW (SBW). Cattle that shipped prior to 12:00 p.m. were adjusted using a 4% shrink, while those shipped after 12:00 p.m. were shrunk by 5%. Railer weights were included in final SBW, though they shipped to a different processing facility and were not factored into any carcass measures. Final SBW and DMI were utilized to determine average daily gain (ADG) and efficiency (G:F).

### Carcass Performance Measures

Steers were shipped to Tyson (Pasco, WA) for harvest based on a projected days on feed and visual assessment of finish. Following removal of the gut mass, livers were scored according to Elanco’s liver abscess classification system by trained personnel. Hot carcass weight (HCW) was recorded after trim. Dressing percentage was considered the percent difference between final SBW and HCW for each pen. USDA quality (QG), yield grades (YG), Certified Angus Beef (CAB) qualifications, and dark cutters were evaluated by a certified USDA grader.

### Statistical Analyses

All statistical analyses, except liver abscess prevalence and type distributions, were performed in R version 4.2.2 (R Core Team, 2021) . Functions from the dplyr package (Wickham et al., 2023a) and purrr package (Wickham and Henry, 2023) were used for data merging and initial calculations. One-way ANOVA analysis was conducted for all quantitative traits. Assumptions of linear models (variance equality and homogeneity, error normality, freedom from outliers, etc.) were first tested using functions from the car package (Fox and Weisberg, 2019) and the rstatix package (Kassambara, 2021). Linear models were constructed using the lmer function of the lme4 package (Bates et al., 2015). From there, functions of the car package (Fox and Weisberg, 2019) developed the ANOVA models. Animals were separated in 5 blocks by arrival date. Block was included in the model as a random effect and was tested using the rand function of the lmerTest package (Kuznetsova et al., 2017). Categorical data distributions were tested by ordinal logistic regression. The initial mixed model was constructed using the clmm function of the ordinal package (Christensen, 2023) and functions of the RVAideMemoire package (Herve, 2023) were utilized for analysis. Estimated marginal means were calculated using the emmeans package of R (Lenth et al., 2023). Liver abscess prevalence and abscess type distributions were tested using the RCBD Stat procedure of SAS (SAS Institute Inc.). Significance was assessed by ANOVA testing of the main effect of treatment; pairwise comparisons were protected by ANOVA significance. Statistical significance was evaluated compared to an α of 0.050. Tendencies were considered when 0.05 < *P* ≤ 0.10. All data visualizations were built in R using the ggplot2 package (Wickham, 2016).

## RESULTS

### Health Responses

Pulls for initial treatment were observed more frequently for NS cattle compared to BD cattle (*P* < 0.01; Table 3). However, NS cattle had a lesser rate of repulls as a proportion of initial pulls (*P* = 0.03). Still, treatment costs did not differ (*P* = 0.81) between groups. Cattle supplemented with NS had fewer (*P* < 0.01) bullers and numerically more railers (*P *= 0.15). Additionally, NS cattle also had a lesser case fatality rate (*P *< 0.01) as a percentage of initial pulls and tended to also have a lesser total mortality rate (*P* = 0.09). However, there was no difference between types of mortality (respiratory, digestive, or other; *P *≥ 0.12). When evaluated by days on feed, BD cattle tended (*P* = 0.08; Table 4) to experience greater mortality during the mid-portion of the feeding period. However, mortality rates did not differ (*P *≥ 0.62) by treatment during the first 45 or the last 90 d on feed.

**Table 3.** Health responses of cattle supplemented with either a direct-fed microbial (BD; 50 mg/hd Bovamine Defend) or a *Saccharomyces cerevisiae* fermentation product (NS; 12 g/hd NaturSafe)

	Treatment			
	BD	NS	SEM^1^	*P* - Value^2^
Morbidity				
First pull, %	21.67	29.43	6.723	< 0.01
Second pull, % of first	16.61	10.08	3.368	0.03
Treatment cost, $/hd	2.36	2.24	0.433	0.81
Case fatality rate, % of first pulls	11.96	6.08	2.241	< 0.01
Total Mortality, %	2.70	1.60	0.513	0.09
Respiratory, %	0.70	0.20	0.264	0.12
Digestive, %	0.43	0.43	0.245	0.99
Other, %	1.50	0.90	0.384	0.22
Bullers, %	2.36	0.86	1.272	< 0.01
Railers, %	0.26	0.70	0.327	0.15

^1^Largest standard error of the mean.

^2^Tested as the ANOVA significance for the main effect.

^a, b^Means without a common superscript are different by pairwise comparison (*P* ≤ 0.05).

^x, y^Means without a common superscript are different by pairwise comparison (*P* ≤ 0.10).

**Table 4.** Mortality by days on feed for cattle supplemented with either a direct-fed microbial (BD; 50 mg/hd Bovamine Defend) or a *Saccharomyces cerevisiae* fermentation product (NS; 12 g/hd NaturSafe)

	Treatment			
	BD	NS	SEM^1^	*P* - Value^2^
Total Mortality, %	2.70	1.60	0.513	0.09
First 45 d on feed, %	0.40	0.30	0.200	0.71
Mid-feeding period, %	1.40	0.60	0.831	0.08
Last 90 d on feed, %	0.90	0.70	0.299	0.62

^1^Largest standard error of the mean.

^2^Tested as the ANOVA significance for the main effect.

^x, y^Means without a common superscript are different by pairwise comparison (*P* ≤ 0.10).

### Performance Responses

Data are reported on a deads-in basis unless otherwise stated. Cattle spent an average of 275 ± 6.2 d on feed (DOF; *P* = 0.71; Table 5). Initial body weight and final shrunk body weight did not differ by treatment [289 and 621 (BD) vs. 288 and 621 (NS) kg; *P *≥ 0.84]. Even though DMI did not differ (*P *= 0.99) by treatment, NS supplemented cattle tended to experience greater ADG (*P *= 0.06). Likewise, NS cattle also benefited from a numeric advantage in efficiency (0.132 vs. 0.130) that was nearing a tendency (*P *= 0.11). When considered deads-out, ADG and G:F did not differ (*P *≥ 0.85) by treatment.

**Table 5.** Feedlot performance of cattle supplemented with either a direct-fed microbial (BD; 50 mg/hd Bovamine Defend) or a *Saccharomyces cerevisiae* fermentation product (NS; 12 g/hd NaturSafe), analyzed on both a deads-in and deads-out basis

	Treatment			
	BD	NS	SEM^1^	*P* - Value^2^
Deads-in basis				
Days on feed	275	275	6.2	0.71
Initial BW, kg	288	288	8.0	0.87
Final SBW, kg	621	620	10.8	0.84
DMI, kg/day	8.85	8.85	0.109	0.99
ADG, kg	1.15^y^	1.17^x^	0.045	0.06
G:F	0.130	0.132	0.0057	0.11
Deads-out basis				
ADG, kg	1.20	1.20	0.040	0.85
G:F	0.135	0.136	0.0055	0.89

^1^Largest standard error of the mean.

^2^Tested as the ANOVA significance for the main effect.

^x,^

^y^Means without a common superscript are different by pairwise comparison (*P *≤ 0.10).

### Carcass Responses

There was no difference (*P* = 0.62; Table 6) for HCW but dressing percentage was greater for NS supplemented cattle (*P *= 0.01). Cattle receiving BD had an almost 2% points advantage (*P *= 0.03) in the proportion of carcasses that graded Prime. There was no difference (*P *≥ 0.15) observed for the percentage of carcasses grading Choice or Select, qualified for Certified Angus Beef (CAB), or dark cutters. Yield grade distribution also did not differ (*P *≥ 0.34) by treatment.

**Table 6.** Carcass performance of steers supplemented with either a direct-fed microbial (BD; 50 mg/hd Bovamine Defend) or a *Saccharomyces cerevisiae* fermentation product (NS; 12 g/hd NaturSafe) after 275 ± 6.2 d on feed

	Treatment			
	BD	NS	SEM^1^	*P* - Value^2^
Hot carcass weight, kg	385.5	387.0	6.60	0.62
Dressing percentage, %	62.04	62.36	0.092	0.01
Quality grade distribution^3^				
Prime, %	5.16	3.18	0.711	0.03
Choice, %	84.92	87.17	1.408	0.15
Select, %	7.93	7.67	1.078	0.83
CAB qualifications, %	26.93	27.72	2.342	0.70
Dark cutters, %	0.86	0.57	0.344	0.43
Yield grade distribution^3^				
USDA YG 1, %	10.34	11.41	1.450	0.45
USDA YG 2, %	55.60	55.75	2.683	0.95
USDA YG 3, %	30.67	29.25	2.464	0.49
USDA YG 4, %	2.63	3.01	0.716	0.61
USDA YG 5, %	0.31	0.10	0.179	0.34

^1^Largest standard error of the mean.

^2^Tested as the ANOVA significance for the main effect.

^3^Tested using ordinal logistic regression.

Liver abscess type and total abscess occurrence were not significantly influenced (*P *≥ 0.19; Table 7) by dietary treatment. However, NS cattle tended to have less A- abscesses (*P *= 0.10).

**Table 7.** Liver abscess prevalence and abscess type of beef × dairy feedlot steers fed a traditional Pacific Northwest finishing diet and supplemented with either a direct-fed microbial (BD; 50 mg/hd Bovamine Defend) or a *Saccharomyces cerevisiae* fermentation product (NS; 12 g/hd NaturSafe)

	Treatment			
	BD	NS	SEM^1^	*P* - Value^2^
Normal, %	57.83	55.78	2.92	0.62
Condemned, %	42.17	44.22	2.92	0.62
A- abscess, %	3.87	1.72	0.79	0.10
A abscess, %	7.75	10.34	1.26	0.19
A + abscess, %	13.83	15.59	2.03	0.56
Adhered, %	5.28	6.43	1.30	0.55
Open abscess, %	4.51	3.21	1.09	0.32
Open and adhered, %	2.41	3.58	1.11	0.48
Distoma, %	4.52	3.35	1.31	0.50

^1^Largest standard error of the mean.

^2^Tested as the ANOVA significance for the main effect.

## DISCUSSION

### Health-Related Outcomes

Supplementation of SCFP has been linked to immune modulation (Jensen et al., 2008; Mahmoud et al., 2020). A study conducted by Rients et al. (2023) reported potential changes in the innate immune system of SCFP-supplemented steers, illustrated by the increased frequency of circulating natural killer cells and activated gamma delta T cells. Burdick Sanchez et al. (2020) suggested that SCFP may “prime” the innate immune system of supplemented calves, allowing for a more rapid response to and resolution of sickness following an LPS challenge. This concept has been addressed by other investigators. In a series of experiments conducted by Cole et al. (1992), a positive performance response was only noted in SCFP-supplemented calves following a viral challenge. While supplementing unchallenged calves did not significantly affect health or performance, morbid calves that received SCFP required fewer days of antibiotic therapy (Cole et al., 1992). Though average treatment days did not differ in Zinn et al. (1999), supplementation of a yeast culture product reduced morbidity and total sick days of newly received calves. Likewise, SCFP inclusion may prepare cattle for pathogen exposure and provide valuable immune support to high-risk or vulnerable individuals (Burdick Sanchez et al., 2020; Rients et al., 2023).

The assertion that SCFP provision may positively influence health and immune status certainly supports results from the present study. While more SCFP-supplemented steers required a health-related pull initially, the rate of repulls amongst this population was lower than those required for DFM-supplemented cattle. There is potential the SCFP product provided immune support for morbid cattle, allowing them to recover more rapidly and with less outside intervention. Additionally, a lesser case fatality rate suggests that fewer morbid cattle supplemented with NS succumbed to their ailment. This is accompanied by a lower total mortality rate, providing further evidence that supports SCFP-modulated improvements in cattle health and immunity. Future evaluations of the mechanism of action behind this particular outcome are warranted. The decreased frequency of bullers in NS cattle is intriguing. To the best of the authors’ knowledge, this outcome has not been previously reported, though it might deserve additional scrutiny.

### Performance Responses

Improvements in cattle health in the present study may have influenced performance results. Increased ADG and a numeric difference in G:F were reported on a deads-in basis. However, when only cattle that remained in their home pen for the entirety of the trial were considered (deads-out), performance did not differ by treatment. Potential benefits to health and a subsequent reduction in removals might account for close-out performance differences more adequately than growth improvements on an individual animal level. Regardless, supplementation with this SCFP product resulted in improved gain and efficiency on a pen level, which is of significant economic importance.

Reported growth performance results associated with SCFP supplementation are variable. For example, experiments evaluating SCFP have reported a negative influence on ADG (Swyers et al., 2014), no impact on performance outcomes (Deters et al., 2018; Huebner et al., 2019), and improved gain and efficiency (Hinman et al., 1998; Wagner et al., 2016; Rients et al., 2023), all on a deads- and removals-out basis. These contradictory results may be due to study design, region, cattle type, diet, product formulation and dose. In the present study, SCFP supplementation resulted in a 1.7% increase in ADG and a 1.6% improvement in gain efficiency, with no observed impact on DMI, on a deads-in basis. However, no performance improvements on a deads-out basis conflicts with other evaluations that evaluate data with mortalities removed. In a meta-analysis by Wagner et al. (2016), dietary SCFP inclusion resulted in a 1% DMI increase and improvements of 6.5% in ADG and 2.6% in G:F relative to a negative control. While greater performance impacts were noted in Wagner et al. (2016), it is important to note that the present study is unique in its direct comparison of SCFP to DFM inclusion, rather than considering either product relative to a negative control. Dietary inclusion of a DFM may independently influence cattle performance, though associated DFM studies are similar to SCFP in the variability of their results (Hanford et al., 2011; Cull et al., 2015; Thompson et al., 2020).

A large-pen study by Scott et al. (2017) comparing the same SCFP and DFM products used in the present experiment reported dissimilar outcomes on a deads- and removals-in basis, with no difference reported in ADG or G:F, but a tendency for SCFP supplementation to increase DMI. The inconsistency between results is not surprising, as Scott et al. (2017) fed the DFM in a positive control diet that also included tylosin and monensin, while the SCFP was supplemented independent of conventional technologies. The authors suggested the DMI response may be attributed to SCFP supplemented heifers not receiving monensin or tylosin, a more stable rumen environment due to SCFP addition, or a change in ruminal kinetics in the DFM supplemented cattle. Regardless, cattle supplemented only SCFP seemed to perform to the same standard as those receiving conventional growth promoting technologies (monensin and tylosin) and a DFM. These results suggest the SCFP product may have a positive influence on growth performance.

The mechanism of action for improvements in efficiency and gain associated with SCFP inclusion have been researched extensively. Research in dairy cattle has reported improved energy utilization of diets supplemented with SCFP (Dann et al., 2000; Erasmus et al., 2005). Supplementation of SCFP may increase the molar proportion of propionate (Hinman et al., 1998) and total VFA production in the rumen (Miller-Webster et al., 2002). Other studies have noted increases in either diet or nutrient digestibility (Wiedmeier et al., 1987; Yoon and Stern, 1996), potentially resulting from the ability of SCFP to help stabilize rumen conditions (Callaway and Martin, 1997). Supplementation of SCFP can also be fed to mitigate digestive disturbance risks that compromise feedlot cattle performance and health (Moya et al., 2009; Wagner et al., 2016).

### Carcass Characteristics

Carcass characteristics of cattle supplemented with a SCFP product vary between experiments. Many studies report no influence of SCFP inclusion on carcass characteristics (Hinman et al., 1998; Scott et al., 2017). Similarly, the present study observed no treatment impact on HCW, the percentage of carcasses that qualified for Certified Angus Beef (CAB) premiums, the rate of carcasses identified as dark cutters, or USDA yield grade distributions. Treatment effects were noted, however, for dressing percentage and the percentage of carcasses that graded USDA Prime. Results from Wagner et al. (2016) differ from present HCW and dressing percentage outcomes. From nine analyzed experiments that evaluated SCFP relative to a negative control, a reported 2.9 kg numeric difference in HCW favored SCFP-supplemented cattle and no difference in dressing percentage was noted. Swyers et al. (2014) actually noted a reduction in HCW, and no dressing percentage change associated with SCFP inclusion. Some early reports of yeast culture feeding reported both heavier HCW and greater dressing percentages, which was attributed to a potential increase in lean content, rather than variation in subcutaneous fat (Nicholson, 1977). Dressing percentage did differ without modification to USDA yield grade distributions in the present study. To accurately judge the source of the variation in dress, however, a more in-depth red meat yield evaluation would be necessary.

Results regarding the influence of SCFP supplementation on USDA quality grade are indeterminate. Several experiments note that SCFP provision has resulted in a greater number of Choice and less Select carcasses (Swyers et al., 2014; Wagner et al., 2016). Conversely, another evaluation observed that marbling scores of cattle supplemented with SCFP in the latter portion of the feeding period were reduced compared to their unsupplemented counterparts (Rients et al., 2023). While reduced marbling scores more closely aligns with present results, it is again important to note the present evaluation was done in comparison to a DFM rather than a negative control. The DFM utilized (Bovamine Defend) did tend to increase the percentage of Choice and Prime carcasses relative to a negative control (Thompson et al., 2020). The greater percentage of Prime carcasses from BD cattle in the present study could be positively attributed to DFM supplementation. However, research has also shown that cattle incurring sickness tend to have lower quality grades ( McNeill, 2001; Anderson and Gleghorn, 2007). With more health pulls and yet less mortality amongst NS supplemented cattle, it stands to reason more previously sick cattle reached slaughter. Likewise, there is potential cattle health may have influenced quality grade differences. Overall, no detrimental impacts of NS supplementation were observed on carcass characteristics.

While a meta-analysis considered liver abscess results from seven studies reported that SCFP inclusion tended to reduce liver abscess rates overall (Wagner et al., 2016), other experiments noted no influence of SCFP supplementation on total liver abscess prevalence (Swyers et al., 2014; Huebner et al., 2019). One previous trial (Scott et al., 2017) noted no difference in liver abscess rate with SCFP, however, that trial was a comparison of the same SCFP product used in the present study versus tylosin (along with monensin plus DFM). Results of present study also suggested treatment did not influence liver abscess type or overall occurrence. In fact, liver abscess rates in this study were similar to the average incidence rate for cattle fed and harvested in the Pacific Northwest (Herrick et al., 2022). Supplementation of NS had no discernible effect on liver abscesses other than a tendency (P = 0.10) for fewer livers with A- abscesses.

### Implications

Supplementation of this SCFP (NaturSafe) improved feedlot cattle health and positively influenced performance when compared to DFM inclusion. Repull rate, case fatality rate, and overall mortality rate were reduced for cattle receiving NaturSafe, potentially influenced by immune modulation capabilities. While these improvements in health did not influence treatment cost, economic advantage might come through associated lot performance improvements. Additional research may be warranted on carcass characteristics of NaturSafe-supplemented cattle—especially concerning whether advantages in dressing percentage could be attributed to additional lean yield. Overall, NaturSafe could be included in feedlot finishing diets of beef × dairy crossbred steers to aid in immune support and attenuate detrimental performance impacts associated with health challenges.
